# Supplementary material for: Ultrastrong and tough paper structure from densified hybrids of multiscale cellulose fibers
Source: Nat Commun. 2026 Mar 13;17:3889. doi: 10.1038/s41467-026-70357-8 (PMC13125482; doi:10.1038/s41467-026-70357-8)
Supplement: Supplementary file 1 — Supplementary Information [file 41467_2026_70357_MOESM1_ESM.pdf]

Supplementary Information for

Ultrastrong and Tough Paper Structure from Densified Hybrids of Multiscale Cellulose Fibers

Liqiong Liao[1], Bingbing Li[1], Zhiping Shi[1], Kai Li[1]*, Yao Lu[1], Yuxin Liu[1], Qi Zhou[2]*

[1] Faculty of Chemical Engineering, Kunming University of Science and Technology, 650500, Kunming, Jingmingnan Road 727, China

[2] Division of Glycoscience, Department of Chemistry, School of Engineering Sciences in Chemistry, Biotechnology and Health, KTH Royal Institute of Technology, AlbaNova University Centre, Stockholm SE-106 91, Sweden.

*Corresponding authors.

Qi Zhou, E-mail: qi@kth.se

Kai Li, E-mail: lkjnkmwh@hotmail.com

This file includes:

Supplementary Fig. 1-14

Supplementary Table 1-4

Supplementary References

Other supplementary materials for this manuscript include the following:

Supplementary Movie 1

Tensile test of a HCP sample prepared from the pulp fiber, microgel, and B-CNF with a composition of 1:1:1 by weight.

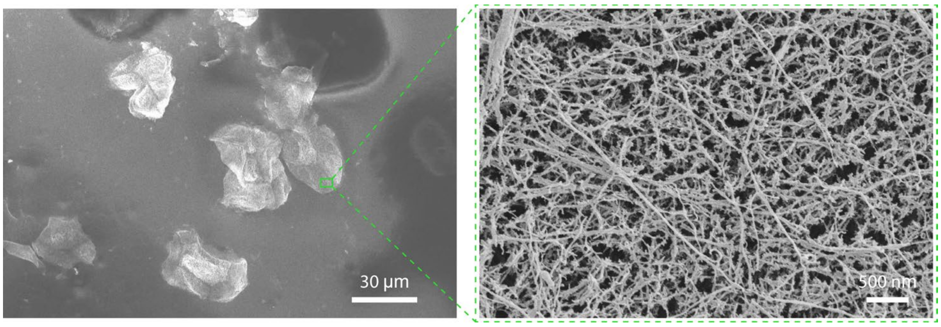

Supplementary Fig. 1 : Morphology of bacterial cellulose microgels.

SEM images showing the micro- and nanoscale structure of bacterial cellulose microgels.

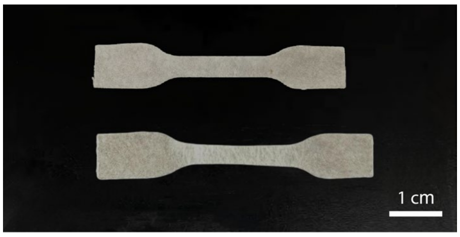

Supplementary Fig. 2 : Deformation of the HCP sample after strain hardening.

Photograph taken after stress removal and before breakage, showing retained plastic deformation.

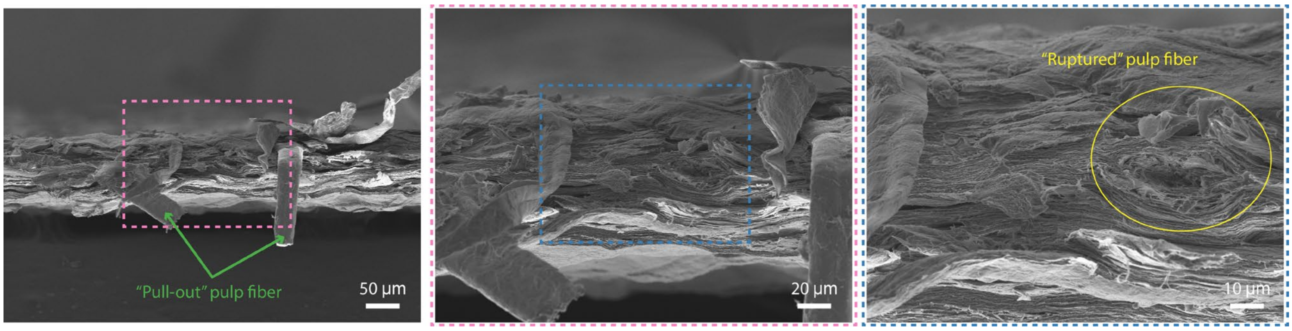

Supplementary Fig. 3 : Tensile fracture surface of the HCP sample.

SEM images showing the fracture surface after tensile testing, highlighting fiber pull-out and fiber rupture.

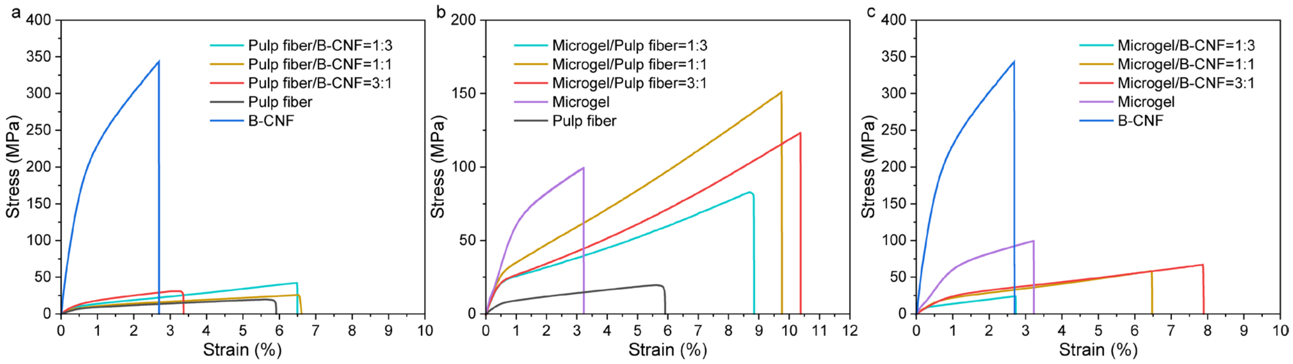

Supplementary Fig. 4 : Tensile stress–strain behavior of binary hybrid papers.

Tensile stress–strain curves of hybrid papers composed of (a) pulp fiber and B-CNF, (b) microgel and pulp fiber, and (c) microgel and B-CNF.

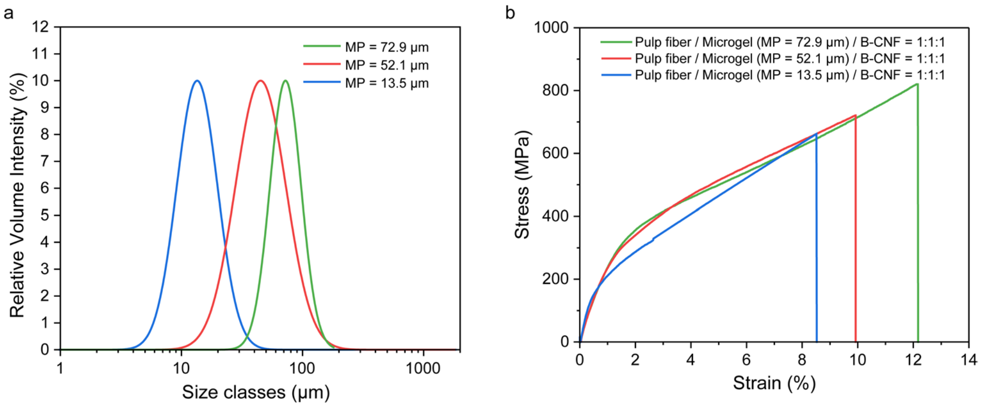

Supplementary Fig. 5 : Effect of microgel size on HCP paper properties.

a Size distribution of the microgels measured by Mastersizer. **b** Tensile stress–strain curves of the HCP papers prepared with microgel with different sizes.

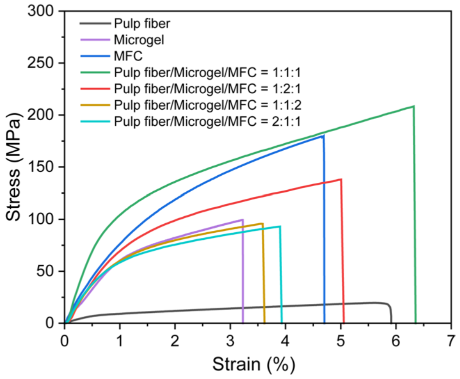

Supplementary Fig. 6 : Effect of MFC on the tensile behavior of HCP papers.

Tensile stress–strain curves of the HCP papers prepared from the pulp fiber, microgel, and MFC with various weight ratios as compared to the papers prepared from the individual components.

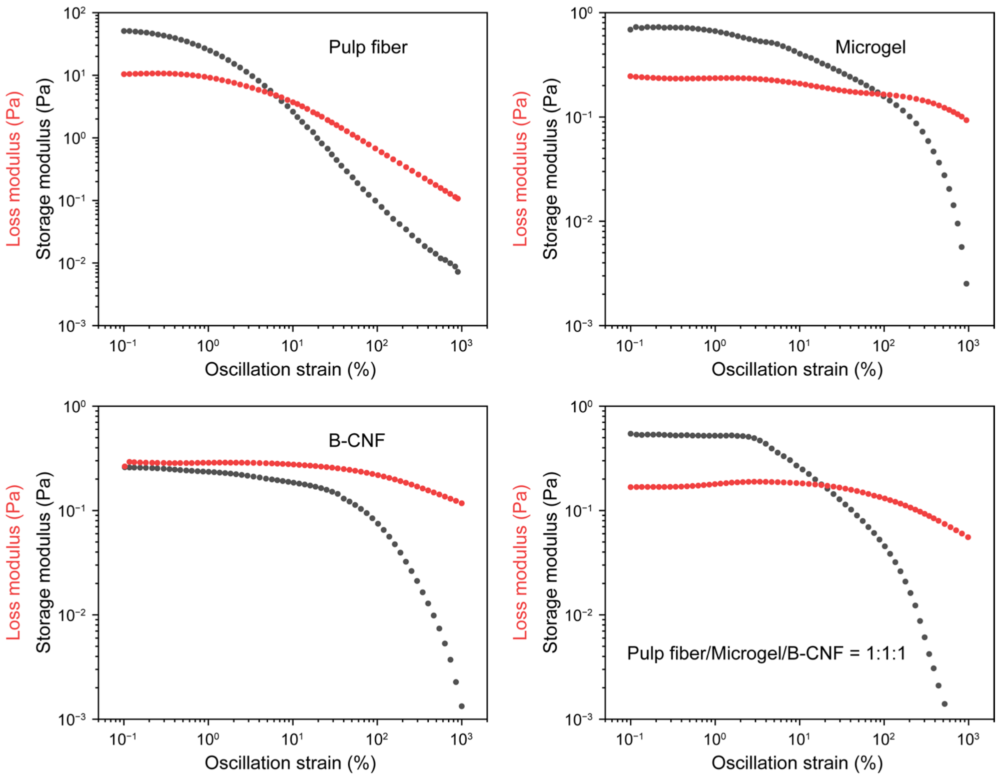

Supplementary Fig. 7 : Rheological behavior of cellulose fiber suspensions.

Dependence of storage modulus and loss modulus on oscillation strain for the 0.2 wt.% water suspensions of neat pulp fibers, neat microgel, neat B-CNF, and the hybridization of the three cellulose fibers. The B-CNF water suspension exhibited a typical sol-like rheological behavior, characterized by a loss modulus that was significantly higher than its storage modulus. In contrast, the pulp fibers and microgel suspensions showed a typical gel-like rheological behavior, indicative of their more structured and elastic nature. Notably, the hybrid suspension composted of pulp fibers, microgel, and B-CNF in a 1:1:1 ratio also exhibited gel-like rheological behavior, as evidenced by a well-defined linear viscoelastic region.

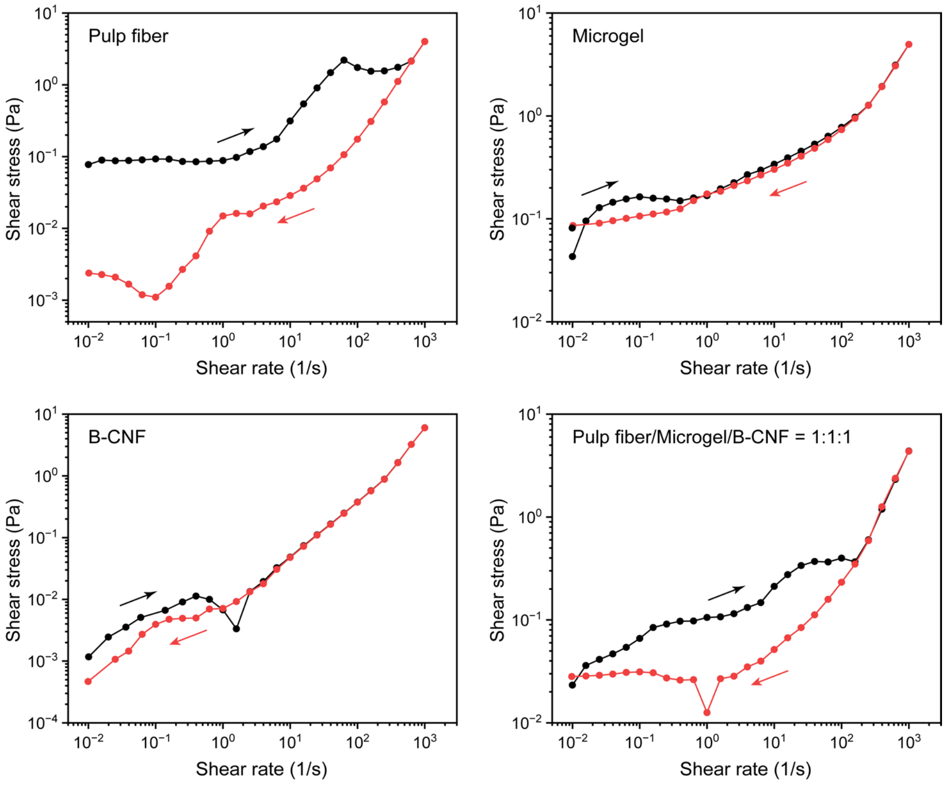

Supplementary Fig. 8 : Thixotropic behavior of cellulose fiber suspensions.

Thixotropic behavior of the 0.2 wt.% water suspensions of neat pulp fibers, neat microgel, neat B-CNF, and the hybridization of the three cellulose fibers. The thixotropic loop test revealed that the hybrid mixture of pulp fibers, microgel, and B-CNF (1:1:1 ratio) generated a larger thixotropic loop compared to the individual components. This observation suggests that the hybrid possesses a more interconnected structure, enabling it to dissipate greater amounts of energy during deformation.

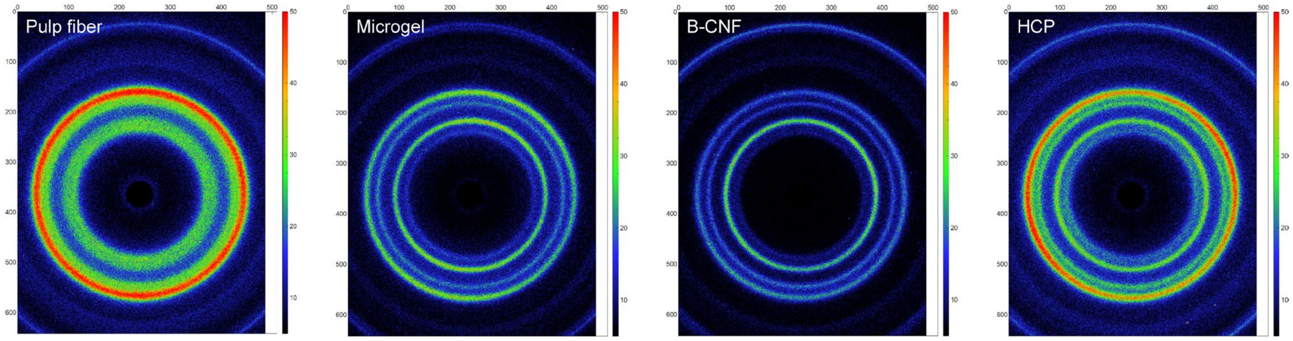

Supplementary Fig. 9 : WAXS diffractograms of HCP and neat cellulose-based papers. Wide-angle X-ray scattering (WAXS) diffractograms collected with the X-ray beam perpendicular to the paper surface for HCP, and for papers prepared from neat pulp fiber (65° SR), neat microgel, and neat B-CNF.

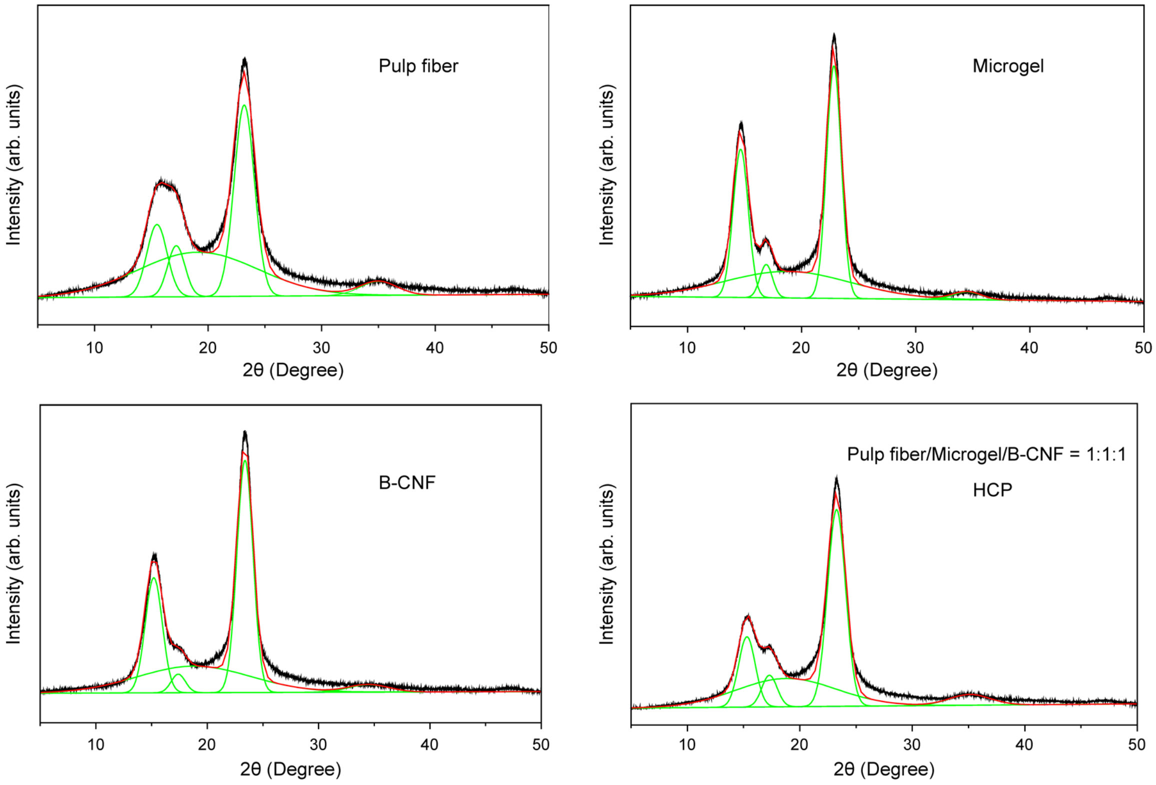

Supplementary Fig. 10 : Determination of cellulose crystallinity by peak deconvolution. X-ray diffraction patterns of pulp fiber (65° SR), microgel, B-CNF, and HCP analyzed using the peak deconvolution method to calculate cellulose crystallinity.

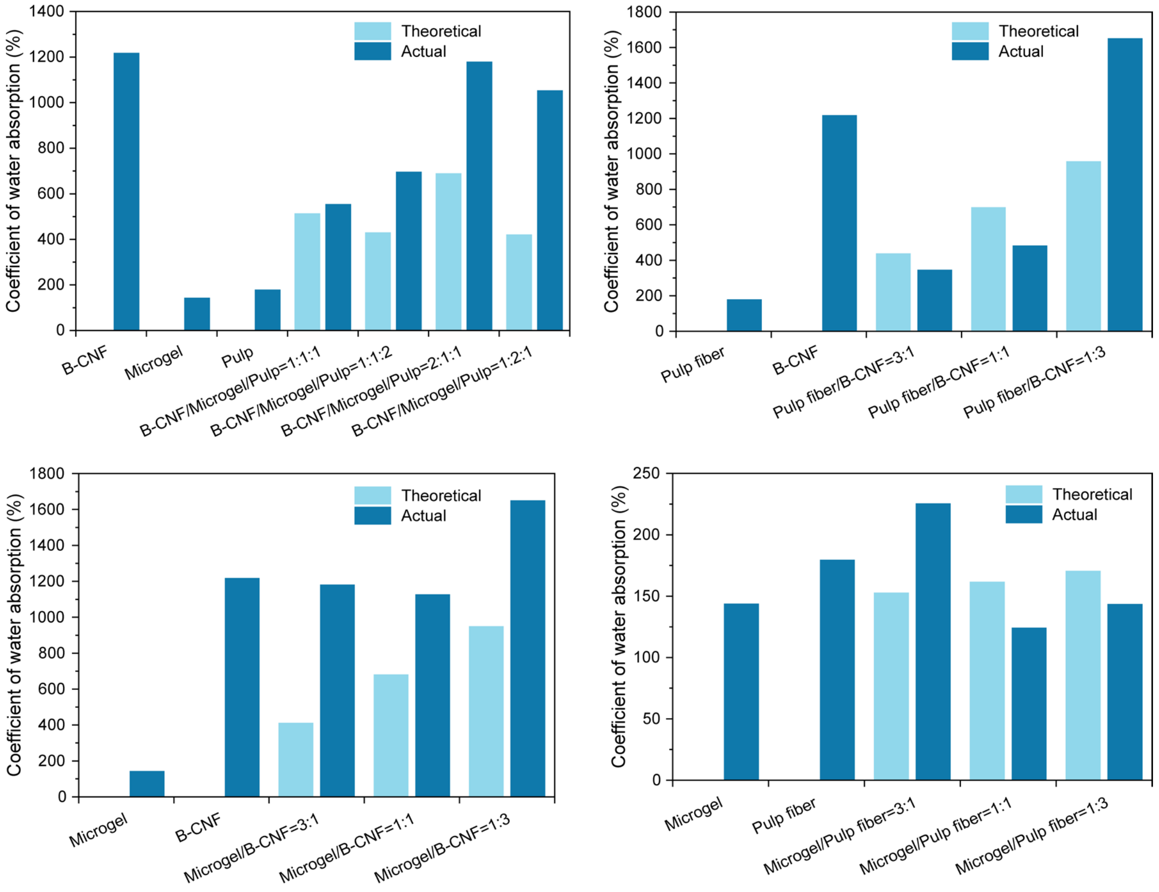

Supplementary Fig. 11 : Water absorption behavior of hybrid cellulose papers. Coefficient of water absorption of papers prepared from various hybrid combinations of pulp fibers, microgel, and B-CNF after incubation in water for 24 h. The theoretical water absorption of each composite paper was calculated as the weighted average of the experimentally measured water absorption values of the individual components (pulp fiber, microgel, and B-CNF), based on their respective mass fractions. The actual water absorption values of all individual components and hybrid papers were determined experimentally.

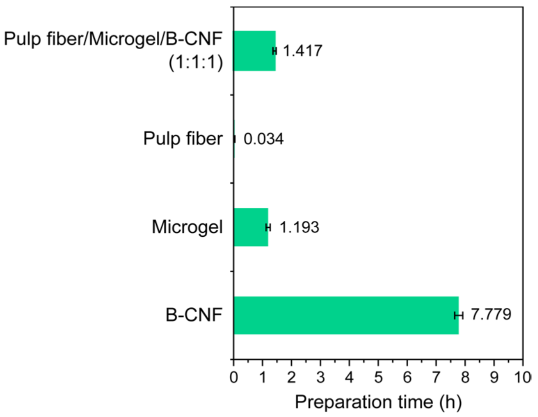

Supplementary Fig. 12 : Vacuum filtration time of HCP and neat fiber-based papers. Vacuum filation time of the HCP paper compared with papers prepared from neat pulp fiber (65° SR), neat microgel, and neat B-CNF. Data are presented as mean values ± standard deviation (SD), based on n = 3 independent replicates.

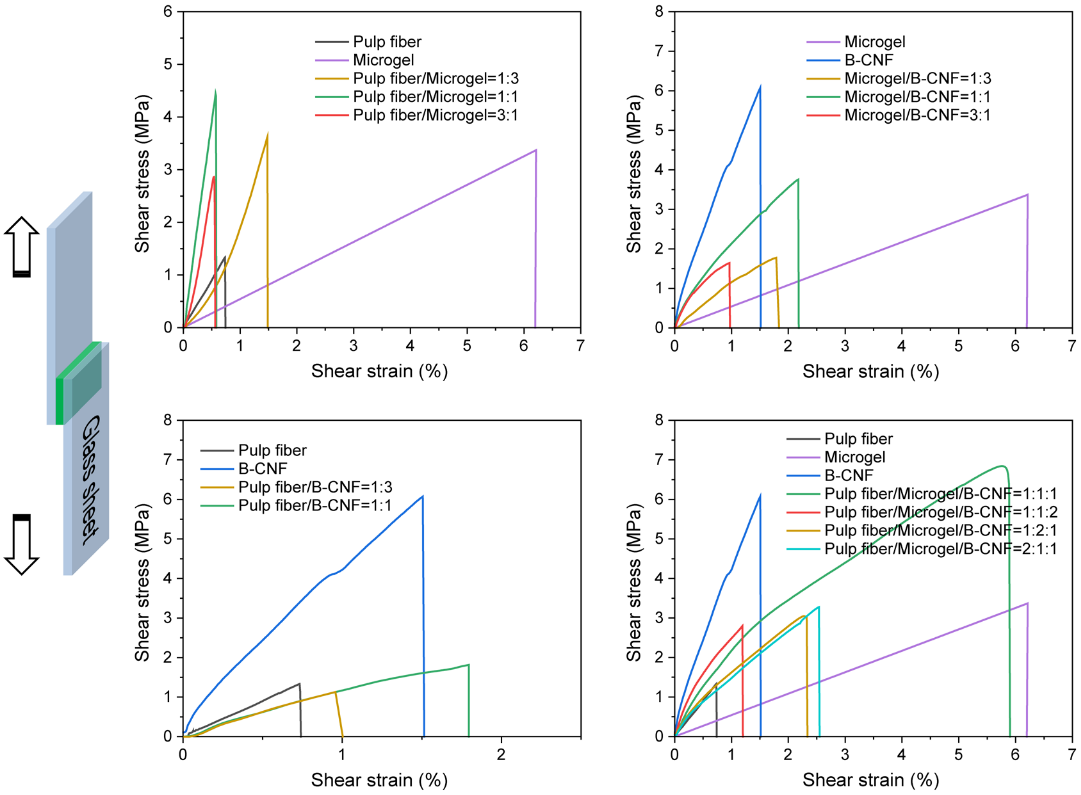

Supplementary Fig. 13 : Comparison of neat, binary, and ternary hybrid cellulose-based adhesive systems on glass substrates.

The lap-shear strength of glass sheetes adhered with dispersions of the pulp fiber (65° SR), microgel, and B-CNF, along with their binary mixtures and ternary mixture.

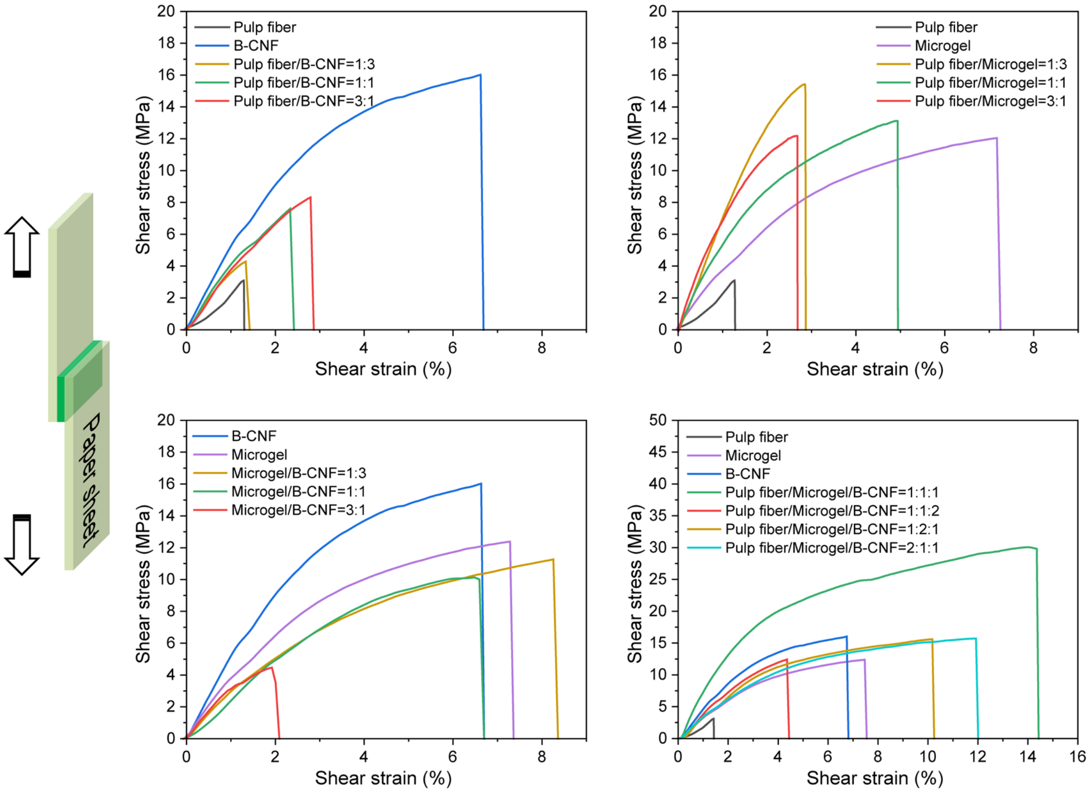

Supplementary Fig. 14 : Comparison of neat, binary, and ternary hybrid cellulose-based adhesive systems on paper substrates.

The lap-shear strength of paper sheetes adhered with dispersions of the pulp fiber (65° SR), microgel, and B-CNF, along with their binary mixtures and ternary mixture.

Supplementary Table 1 Mechanical properties of the hybrid cellulose paper (HCP) developed in this work and different studies correlated with the Ashby plot in Fig. 1f.

Sample	Tensile Strength (MPa)	Young's Modulus (GPa)	Strain at failure (%)	Density (g/cm^3)	Specific tensile strength ($MPa\ cm^3/g$)	Spcific Young's Modulus ($GPa\ cm^3/g$)	Toughness (MJ/m^3)
Random oriented (isotropic) cellulose papers or films							
Regenerated cellulose[1]	106 [a]	4.3	12.4	1.245	85.1	3.45	9.8
BC film[2, 3]	194 [b]	11.4	3.4	0.64	303	17.8	3.8
TEMPO-oxidized BC[4]	102 [a]	18.5	0.9	1.19	85.7	15.5	0.6
Enz-CNF[5]	214 [c]	13.2	10.1	1.08	198	12.2	15
Lignin-rich MFC[6]	283 [c]	15.1	7.4	1.39	204	10.9	- [e]
Holo-CNF[7]	320 [c]	21.0	4.8	1.47	218	14.3	9.8
TEMPO-CNF[8]	321 [d]	17	9	1.41	228	12.1	16
MFC/wood fiber[9]	160 [c]	10.1	4.2	0.97	165	10.4	4.4
HCP (this work)	811 [d]	36.1	11.4	1.10	737	32.8	56.5
Oriented or stretched (anisotropic) cellulose papers and films							
Denlignified wood film[10]	449 [c]	51.1	1.6	1.32	340	38.7	- [e]
Regenerated cellulose[1]	253 [a]	14.6	16.5	1.245	203	11.7	41.1
BC film[2]	1005 [b]	48.1	4.4	1.18	852	40.8	24.7

[a] Tensile test condition: data not reported.

[b] Tensile test condition: relative humidity (RH) of 40% and Temperature (T) of 23 ^{o}C.

[c] Tensile test condition: RH of 50% and T of 23 ^{o}C.

[d] Tensile test condition: RH of 50% and T of 25 ^{o}C.

[e] Data not reported.

Supplementary Table 2 Tensile mechancial properties of the different hybrid papers presented in Fig. 1e, 1g, and 1h, Fig. 3c and 3d.

Samples [a]	Pulp fiber (SR)	Tensile Strength (MPa)	Strain at failure (%)	Young's Modulus (GPa)	Toughness (MJ/m^3)
Pulp fiber	65°	17.9±1.5	5.9±1.0	1.3±0.3	0.6±0.2
Microgel	-	102.3±7.2	3.6±0.4	5.5±0.7	2.7±1.1
B-CNF	-	327.4±15.9	2.2±0.4	20.6±2.9	4.7±0.7
HCP (1:1:1)	65°	811.0±17.1	11.4±1.0	36.1±1.8	56.5±6.1
HCP (1:1:1.2)	65°	691.2±4.0	6.6±1.1	23.7±1.0	25.5±1.6
HCP (1.2:1:1)	65°	665.2±5.0	6.2±0.7	21.3±1.0	29.1±1.3
HCP (0.8:1:1)	65°	457.2±8.0	4.8±0.4	19.9±0.6	14.6±1.0
HCP (1:1:0.8)	65°	440.6±8.9	6.2±0.5	15.6±0.9	17.6±1.0
HCP (1:1:2)	65°	38.5±3.1	9.0±1.3	3.1±0.7	2.2±0.4
HCP (1:2:1)	65°	77.8±9.1	13.2±1.3	2.9±0.2	6.6±1.1
HCP (2:1:1)	65°	55.2±3.7	12.9±3.4	1.3±0.2	4.7±0.6
HCP (1:1:1)	15°	344.2±26.1	4.0±0.5	17.0±2.0	9.3±1.2
HCP (1:1:1)	25°	437.4±27.0	4.5±1.0	22.2±2.8	13.8±1.6
HCP (1:1:1)	35°	541.4±9.0	5.5±0.5	26.0±2.6	19.0±1.8
HCP (1:1:1)	45°	585.8±14.2	5.8±0.8	29.7±0.7	22.4±1.3
HCP (1:1:1)	55°	623.7±20.5	7.5±0.5	30.5±3.3	30.7±2.0
HCP (1:1:1)	65°	811.0±17.1	11.4±1.0	36.1±1.8	56.5±6.1
HCP (1:1:1)	75°	531.8±27.2	12.0±1.0	12.5±2.2	34.4±3.8
HCP (1:1:1)					
Natural drying	65°	811.0±17.1	11.4±1.0	36.1±1.8	56.5±6.1
Completely drying	65°	157.3±15.9	5.3±1.1	8.5±0.5	4.4±0.8
RH 70%	65°	370.7±4.8	16.0±0.6	17.6±0.5	7.0±0.06
RH 90%	65°	168.0±6.7	19.9±0.8	9.4±0.7	9.2±0.3

[a] The riatio in the parenthesis for each sample indictate the weight ratio of Pulp fiber, Microgel, and B-CNF.

Supplementary Table 3 Densities and porosities of the papers of the HCP paper as compared to the papers prepared from neat pulp fiber (65° SR), neat microgel, and neat B-CNF.

Paper samples	Bulk density[a] (g/cm³)	Porosity[b] (%)
Pulp fibers	0.66	56
B-CNF	1.11	26
Microgel	1.15	23
Pulp fiber/B-CNF=1:1	0.84	44
Pulp fiber/Microgel=1:1	0.79	47
HCP - Pulp fiber/Microgel/B-CNF=1:1:1	1.10	27

[a] Bulk density of the cellulose films was measured using a fully automated pressed-mercury porosimeter (Autopore V 9505, Micromeritics, USA).

[b] Porosity was calculated according to the equation (1), where ρ_{bulk} corresponds to the bulk density of the paper and $\rho_{cellulose}$ corresponds to the density for the cellulose, which is assumed to be 1.5 g/cm^3.

$$Porosity(\%) = \left(1 - \frac{\rho_{bulk}}{\rho_{cellulose}}\right) \times 100 \qquad (1)$$

Supplementary Table 4 Filtration time and mechanical properties of the HCP paper from this work as compared to the cellulose films prepared from TEMPO-CNF,[8] Holo-CNF,[7] and Enz-CNF[5] as reported in literature.

	HCP	TEMPO-CNF[8]	Holo-CNF[7]	Enz-CNF[5]
Filtration time (h)	< 1.5	~ 24	< 2.5	< 2.5
Thickness (μm)	70	30–40	35	60–80
Tensile strength (MPa)	811	321	320	214
Tensile modulus (GPa)	36.1	17	21	13.2
Strain at break (%)	11.4	9	4.8	10.1
Toughness (MJ/m^3)	56.5	16	9.8	15
Specific strength (MPa cm^3/g)	737	228	218	198
Specific modulus (GPa cm^3/g)	32.8	12.1	14.3	12.2

Supplementary References

1. Ye D*, et al.* Ultrahigh Tough, Super Clear, and Highly Anisotropic Nanofiber-Structured Regenerated Cellulose Films. *ACS Nano* **13**, 4843-4853 (2019).
2. Wang S*, et al.* Transparent, Anisotropic Biofilm with Aligned Bacterial Cellulose Nanofibers. *Adv. Funct. Mater.* **28**, 1707491 (2018).
3. Retegi A*, et al.* Bacterial cellulose films with controlled microstructure–mechanical property relationships. *Cellulose* **17**, 661-669 (2010).
4. Yang KY, Wloch D, Lee KY. TEMPO-oxidised nanocellulose hydrogels and self-standing films derived from bacterial cellulose nanopaper. *RSC Adv.* **11**, 28352-28360 (2021).
5. Henriksson M, Berglund LA, Isaksson P, Lindström T, Nishino T. Cellulose nanopaper structures of high toughness. *Biomacromolecules* **9**, 1579-1585 (2008).
6. Li H, Kulachenko A, Mathew AP, Stoltz RB, Sevastyanova O. Enhancing the Strength and Flexibility of Microfibrillated Cellulose Films from Lignin-Rich Kraft Pulp. *ACS Sustainable Chem. Eng.* **11**, 16793-16805 (2023).
7. Yang X, Reid MS, Olsén P, Berglund LA. Eco-Friendly Cellulose Nanofibrils Designed by Nature: Effects from Preserving Native State. *ACS Nano* **14**, 724-735 (2020).
8. Benítez AJ, Walther A. Counterion Size and Nature Control Structural and Mechanical Response in Cellulose Nanofibril Nanopapers. *Biomacromolecules* **18**, 1642-1653 (2017).
9. Sehaqui H, Allais M, Zhou Q, Berglund LA. Wood cellulose biocomposites with fibrous structures at micro- and nanoscale. *Compos Sci Technol* **71**, 382-387 (2011).
10. Li K, Wang S, Chen H, Yang X, Berglund LA, Zhou Q. Self-Densification of Highly Mesoporous Wood Structure into a Strong and Transparent Film. *Adv. Mater.* **32**, 2003653 (2020).
