# Supplementary material for: Ultrastrong and tough paper structure from densified hybrids of multiscale cellulose fibers
Source: Nat Commun. 2026 Mar 13;17:3889. doi: 10.1038/s41467-026-70357-8 (PMC13125482; doi:10.1038/s41467-026-70357-8)
Supplement: Supplementary file 2 — Description of Additional Supplementary Files [file 41467_2026_70357_MOESM2_ESM.pdf]

Supplementary Movie 1:

Tensile test of a HCP sample prepared from the pulp fiber, microgel, and B-CNF with a composition of 1:1:1 by weight.
